# 605. Efficacy and pharmacokinetics-pharmacodynamics of ATI-2307 in a rabbit model of cryptococcal meningoencephalitis

**DOI:** 10.1093/ofid/ofac492.657

**Published:** 2022-12-15

**Authors:** Charles D Giamberardino, Jennifer L Tenor, Dena Toffaletti, Julia R Palmucci, Don Cilla, William Hope, John R Perfect

**Affiliations:** Duke University, Durham, North Carolina; Duke University, Durham, North Carolina; Duke University Medial Center, Durham, North Carolina; Duke University, Durham, North Carolina; Appili Therapeutics, Frederick, Maryland; University of Liverpool, Liverpool, England, United Kingdom; Duke University, Durham, North Carolina

## Abstract

**Background:**

Cryptococcal meningitis (CM) is a fungal disease with significant global morbidity and mortality. There are multiple new therapies being developed to improve the current regimens involving amphotericin B, flucytosine, and fluconazole. ATI-2307 is a novel arylamidine which inhibits mitochondrial function in yeasts. We tested ATI-2307 in a rabbit model of CM and evaluated its efficacy, pharmacokinetics, and pharmacodynamics (PK-PD).

**Methods:**

Immune suppressed rabbits were infected with Cryptococcus neoformans. Starting two days after the infection, they were treated with ATI-2307 (1, 2, or 3 mg/kg), amphotericin B (1 mg/kg), fluconazole (80 mg/kg), a combination of ATI-2307 (1 mg/kg) and fluconazole (80 mg/kg), or they were untreated. ATI-2307 was given daily subcutaneously. Fungal burden and drug levels were quantified in the CSF during the infection and in the brain tissue at study termination, day 4, 10, or 14 post infection. Drug pharmacokinetics in the blood were examined on day 8 post infection. Drug pharmacokinetics in the CSF and brain tissue were examined at day 4 post infection.

**Results:**

Daily dosing with ATI-2307 reduced the yeast burden in both the cerebrospinal fluid (CSF) and brain tissue compared to controls. Furthermore, when combined with fluconazole, ATI-2307 reduced the CSF fungal burden below the limit of detection. In fact, we observed a large reduction in fungal burden relative to untreated controls with only three doses, which was sustained for 1 week after the cessation of dosing. The kinetics of ATI-2307 indicated that the drug has a long gamma phase in both blood and CSF. In addition, our analysis of the drug levels in the brain tissue indicated that it dramatically accumulated in the meninges, which may then serve as a site of sustained release.

Efficacy of ATI-2307 on CSF Fungal Burden and Distribution of ATI-2307 in Brain Tissue

**Figure:**
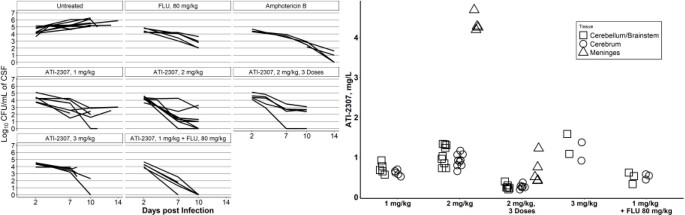

ATI-2307 was dosed daily starting at day 2 post infection through the study endpoint, day 10 or 14 post infection, with the exception of the 2 mg/kg, 3 dose group, which only received doses on days 2, 3, and 4 post infection.

**Conclusion:**

Taken together, these data provide evidence for ATI-2307 as a promising therapy for CM and it may require fewer doses than existing treatments and provide additive anticryptococcal activity with azole treatment.

**Disclosures:**

**Charles D. Giamberardino, Jr., MR**, Affimed NV: Stocks/Bonds|Appili Therapeutics: Grant/Research Support|Interventional Analgesix Inc: Grant/Research Support|Minnetronix: Grant/Research Support|Pfizer: Grant/Research Support|Quoin Pharmaceuticals: Stocks/Bonds|Sfunga: Grant/Research Support **Jennifer L. Tenor, PhD**, Appili: Grant/Research Support|Pfizer: Grant/Research Support **Dena Toffaletti, PhD**, Appili Therapeutics: Grant/Research Support|Pfizzer: Grant/Research Support **Don Cilla, Pharm.D., M.B.A.**, Appili Therapeutics: Employee|Appili Therapeutics: Stocks/Bonds **William Hope, OBE (FRACP, FRCPA, PhD)**, Antabio: Grant/Research Support|Appili Therapeutics: Advisor/Consultant|BioVersys: Grant/Research Support|Bugworks: Grant/Research Support|Centauri: Advisor/Consultant|F2G: Advisor/Consultant|F2G: Grant/Research Support|NAEJA-RGM Pharmaceuticals Inc: Advisor/Consultant|NAEJA-RGM Pharmaceuticals Inc: Grant/Research Support|National Institute of Health Research (NIHR).: Co-lead for Infectious Diseases|Pfizer: Advisor/Consultant|Pfizer: Grant/Research Support|Phico Therapeutics: Grant/Research Support|Specialist Advisory Committee for GARDP: Advisor/Consultant|Spero Therapeutics: Advisor/Consultant|Spero Therapeutics: Grant/Research Support|The Global Antibiotic Research and Development Partnership (GARDP): Grant/Research Support|VenatoRx: Advisor/Consultant **John R. Perfect, MD**, Appili Therapeutics: Advisor/Consultant|Appili Therapeutics: Grant/Research Support|Astellas: Advisor/Consultant|Astellas: Grant/Research Support|Matinas: Advisor/Consultant|Merck: Advisor/Consultant|Pfizer: Advisor/Consultant|Pfizer: Grant/Research Support|Scynexis: Advisor/Consultant.

